# Knowledge of the Importance of Folic Acid Among Women of Childbearing Age in the United Arab Emirates: A Cross-Sectional Study

**DOI:** 10.7759/cureus.103913

**Published:** 2026-02-19

**Authors:** Hazim I Al Sane, Mariam H Hussein, Faizeh H Jadalhaq, Shrouq S Al-Ahmad, Ahmed K Alhammadi, Fatima A Almuaini, Amal Hussein, Mohamed A Saleh

**Affiliations:** 1 College of Medicine, University of Sharjah, Sharjah, ARE; 2 Family and Community Medicine, University of Sharjah, Sharjah, ARE

**Keywords:** folate, folic acid, neural tube defects (ntds), ntd, public awareness

## Abstract

Background: Adequate folic acid intake before and during early pregnancy is essential for the prevention of neural tube defects (NTDs). Despite established recommendations, gaps in knowledge and appropriate use of folic acid persist among women of reproductive age.

Objective: This study aimed to assess the level of knowledge regarding folic acid and its role in pregnancy among women of childbearing age in the United Arab Emirates (UAE) and to identify factors associated with better awareness.

Methods: A cross-sectional study was conducted among women aged 18-49 years residing in the UAE using a self-administered online questionnaire distributed via social media platforms. Non-probability sampling methods, including snowball and voluntary sampling, were employed. Data were analyzed using IBM SPSS Statistics for Windows, V. 28.0 (IBM Corp., Armonk, NY, USA). Descriptive statistics were used to summarize responses, and associations between knowledge levels and sociodemographic variables were examined using chi-squared tests and Spearman's correlation.

Results: A total of 379 women participated (median age: 31 years). Overall awareness of folic acid was high (333/379, 87.9%); however, only 214/379 (56.5%) correctly identified its role in preventing NTDs, and 147/379 (38.7%) recognized that supplementation should begin before conception. Higher educational attainment was significantly associated with better knowledge of folic acid (Spearman's r = 0.219; p = 0.001), while no significant association was found between knowledge level and previous pregnancy history (p = 0.124). Healthcare providers were the most commonly reported source of information (271/379, 71.5%).

Conclusion: Although awareness of folic acid among women of childbearing age in the UAE is relatively high, important gaps remain regarding its preventive role and the optimal timing of supplementation. Strengthening preconception education through healthcare services and public health initiatives is essential to improve maternal and fetal health outcomes.

## Introduction

Folic acid, the synthetic form of folate (vitamin B9), is essential for DNA synthesis, cellular division, and methylation processes, all of which are critical during early embryonic development [1]. Insufficient folic acid intake in the periconceptional period has been strongly associated with neural tube defects (NTDs), including spina bifida and anencephaly, as well as other congenital anomalies such as orofacial clefts and congenital heart defects [2]. In addition to its role in fetal development, adequate folic acid intake has been linked to improved maternal outcomes, including reduced risks of preeclampsia, preterm birth, and low birth weight [3].

Because neural tube closure occurs within the first four weeks of pregnancy, often before pregnancy recognition [1], folic acid supplementation should begin prior to conception and continue through early pregnancy. Nevertheless, evidence suggests that many women of childbearing age either lack adequate knowledge regarding folic acid or initiate supplementation too late to achieve optimal preventive benefit [2].

In the United Arab Emirates (UAE), congenital anomalies remain a significant contributor to infant morbidity and mortality. A recent national study reported approximately 704 major congenital anomalies per 10,000 live births, including structural and chromosomal anomalies, rather than NTDs alone [4]. While folic acid supplementation is specifically known to reduce the risk of NTDs and certain other congenital anomalies, it does not prevent all types of congenital conditions. Previous studies conducted in the UAE and neighboring countries have reported high general awareness of folic acid but limited understanding of its specific preventive role and correct timing of use, with sociodemographic characteristics, health literacy, and access to reliable health information significantly influencing women's awareness and adherence to recommended supplementation practices [5-9]. However, most prior UAE-based studies have been limited to specific subpopulations, single institutions, or smaller regional samples, and few have comprehensively examined both knowledge levels and associated sociodemographic predictors across a broader cohort of women of reproductive age. The present study addresses this gap by providing updated, population-based data from a diverse sample of women residing in the UAE and by evaluating the factors associated with higher knowledge levels.

Given the UAE's diverse population and evolving healthcare landscape, understanding current knowledge levels among women of reproductive age is vital for informing effective public health strategies. This study aimed to assess awareness and knowledge of folic acid among women of childbearing age (18-49 years) in the UAE and to examine the sociodemographic factors and sources of information associated with better understanding.

## Materials and methods

Study design

A cross-sectional study was conducted at the University of Sharjah, UAE, and responses were collected online via social media platforms (WhatsApp, Facebook, Instagram) to assess knowledge and awareness of folic acid among women of childbearing age residing in the UAE.

Study population and sample size

Women aged 18-49 years living in the UAE were eligible to participate. Data were collected between February and April 2023 using an online structured questionnaire (see Appendices). Participants who were unable to read Arabic or English were excluded, as the questionnaire was available only in these languages. A total of 379 participants completed the survey and were included in the final analysis. The survey platform required all mandatory fields to be completed before submission; therefore, no incomplete responses were recorded. To minimize duplicate entries, participants were asked to provide the first three letters of their first and last names for screening purposes, and no duplicate responses were identified.

Data collection and study measures

Data were collected using a self-administered online questionnaire distributed via social media platforms. Non-probability sampling methods, including voluntary and snowball sampling, were used to recruit participants.

The questionnaire consisted of 18 items divided into three sections: (1) sociodemographic characteristics (age, nationality, marital status, educational attainment, occupation, monthly family income, access to health insurance, and pregnancy history); (2) awareness and knowledge of folic acid, including prior awareness (having heard of folic acid), benefits, preventive role in NTDs, and timing of supplementation; and (3) sources of information regarding folic acid.

Ethics statement

The study adhered to ethical standards and received approval from the University of Sharjah Research Ethics Committee (approval number: REC-23-02-23-02-PG). Participation was voluntary, and informed consent was implied through completion of the survey. Participants were informed that they could discontinue the survey at any time prior to submission without consequence. However, once the questionnaire was submitted, responses could not be withdrawn, as data were collected anonymously and were not linked to personally identifiable information. No directly identifiable personal information (such as full names, identification numbers, or contact details) was collected. Participants were asked to provide only the first three letters of their first and last names solely for the purpose of screening for duplicate responses. These partial identifiers were not used for identification and were stored securely with restricted access. All data were anonymized prior to analysis to ensure confidentiality.

Questionnaire and knowledge score assessment

The questionnaire was developed by adapting items from previously published and validated instruments assessing knowledge and practices related to folic acid supplementation in similar populations. The items were modified to ensure contextual relevance to the UAE population and were reviewed by the research team to establish face and content validity prior to distribution. The primary outcome of the study was the folic acid knowledge score. Awareness was defined separately as having previously heard of folic acid (yes/no), while knowledge was assessed using a composite scoring system. Knowledge was evaluated using structured items within the "Knowledge and Attitudes" section of the questionnaire. The three knowledge items included the following: (1) the appropriate timing of folic acid supplementation, (2) the target population for supplementation (women planning pregnancy and pregnant women), and (3) the main preventive benefit of folic acid during pregnancy (prevention of NTDs). Correct responses to these knowledge-based questions were assigned 1 point, while incorrect or "I do not know" responses were assigned 0 points. The total knowledge score ranged from 0 to 3. Participants scoring ≥2 were categorized as having good knowledge, while those scoring ≤1 were categorized as having poor knowledge. Missing responses were treated as incorrect and assigned a score of 0.

Statistical analysis

Data were analyzed using IBM SPSS Statistics for Windows, V. 28.0 (IBM Corp., Armonk, NY, USA). Descriptive statistics were used to summarize participant characteristics and responses and were reported as frequencies and percentages for categorical variables and medians with interquartile ranges for continuous variables. The primary outcome, folic acid knowledge, was analyzed both as a categorical variable (good vs poor knowledge) and as a continuous ordinal score (0-3). Associations between categorical knowledge level and sociodemographic variables were examined using chi-squared tests. When knowledge was treated as a continuous ordinal score, nonparametric methods were applied, and Spearman's rank correlation coefficient (ρ) was used to assess correlations between educational level and knowledge score. A p-value of <0.05 was considered statistically significant.

## Results

Data were collected between February and April 2023. A total of 379 women completed the survey and were included in the final analysis. The online questionnaire required completion of all mandatory fields prior to submission; therefore, no incomplete responses were recorded, and the completion rate among those who initiated the survey was 100%. The median age of participants was 31 years (interquartile range (IQR): 29-33 years). The majority were non-UAE nationals (238/379, 62.8%), while 141/379 (37.2%) were UAE nationals. Regarding marital status, 211/379 (55.6%) were married, 159/379 (41.9%) were unmarried, and 9/379 (2.3%) were divorced or widowed. More than half of the participants reported having experienced at least one pregnancy (203/379, 53.4%), whereas 170/379 (45%) had never been pregnant, and 6/379 (1.6%) did not disclose their pregnancy history. Regarding monthly family income, 37/379 (9.8%) reported earning less than 5,000 AED, 101/379 (26.6%) reported 5,000-19,999 AED, 128/379 (33.8%) reported 20,000-30,000 AED, and 113/379 (29.8%) reported more than 30,000 AED. The majority of participants (296/379, 78.1%) reported having access to health insurance, while 83/379 (21.9%) did not. No additional missing data were observed for other study variables.

Sociodemographic characteristics

The majority of participants had attained a college-level education or higher, and a substantial proportion were employed in professional or administrative roles. A detailed summary of participants' educational levels, occupations, and other sociodemographic characteristics is presented in Table 1.

**Table 1:** Sociodemographic characteristics of the study participants (n = 379). IQR: interquartile range; UAE: United Arab Emirates; AED: United Arab Emirates Dirham

Characteristic	n (%)
Total participants	379 (100%)
Age (median, IQR)	31 years (IQR: 29-33)
Nationality	
UAE national	141 (37.2%)
Non‐UAE national	238 (62.8%)
Marital status	
Married	211 (55.6%)
Unmarried	159 (41.9%)
Divorced/widowed	9 (2.3%)
Pregnancy history	
At least one pregnancy	203 (53.4%)
Never pregnant	170 (45%)
Not disclosed	6 (1.6%)
Education	
Secondary/preparatory	37 (9.8%)
College/university	277 (73%)
Postgraduate	65 (17.2%)
Occupation	
Professional/administrative	168 (44.3%)
Student	99 (26.1%)
Homemaker	71 (18.7%)
Unemployed/other	41 (10.9%)
Family income	
<5,000 AED	37 (9.8%)
5,000-19,999 AED	101 (26.6%)
20,000-29,999 AED	128 (33.8%)
>30,000 AED	113 (29.8%)
Health insurance access	
Yes	296 (78.1%)
No	83 (21.9%)

Knowledge and awareness of folic acid

Overall, 333/379 participants (87.9%) reported having heard of folic acid, which was defined as the awareness variable in this study. All participants, regardless of awareness status, completed the subsequent knowledge-related questions. However, variation was observed in the depth of knowledge. When asked about its primary role, 214/379 (56.5%) correctly identified its function in preventing NTDs. In contrast, 82/379 (21.6%) believed folic acid was mainly important for general maternal health, while 49/379 (12.9%) were unsure of its purpose, and the remaining participants selected other incorrect responses.

The median knowledge score was 2 (IQR: 1-3). Overall, 197 participants (52%) demonstrated good knowledge (score ≥2), whereas 182 (48%) had poor knowledge (score ≤1).

**Figure 1:**
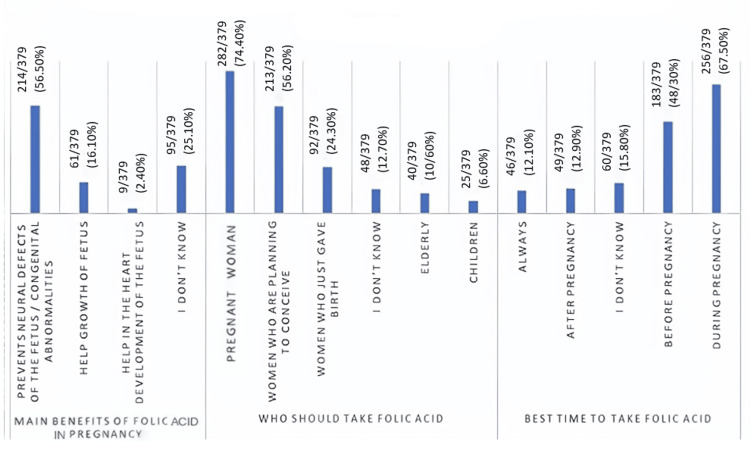
Women's responses to knowledge items related to folic acid (n = 379).

Knowledge regarding the appropriate timing of supplementation was limited. Only 147/379 participants (38.7%) correctly recognized that folic acid should be initiated before conception, whereas 186/379 (49.2%) believed it should be started only after pregnancy was confirmed (Figure 1).

Sources of information

**Figure 2:**
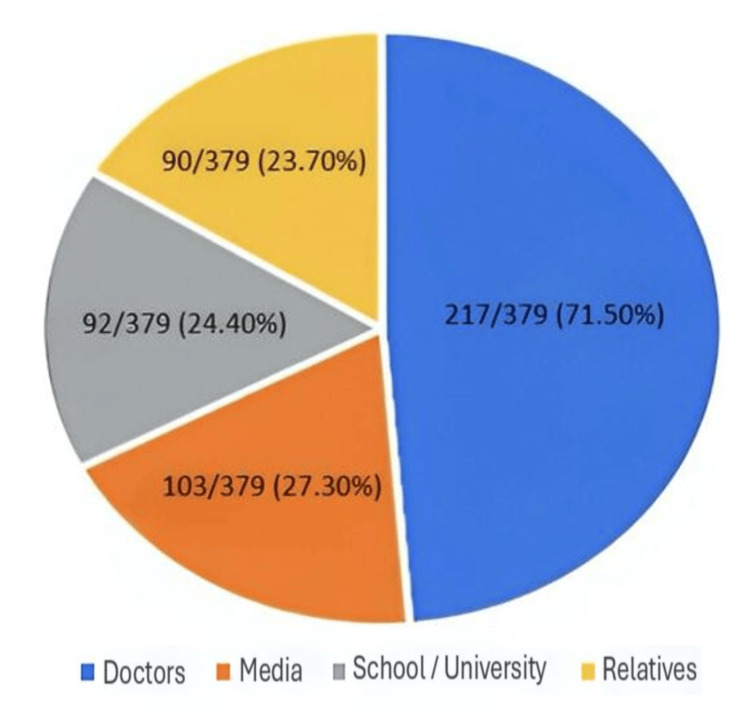
Sources of information regarding folic acid among women of childbearing age (n = 379).

Doctors and other healthcare providers were the most frequently reported source of folic acid information (217/379, 71.5%). Media sources, including television, radio, and online platforms, were cited by 103/379 participants (27.3%), followed by educational institutions (92/379, 24.4%) and relatives or friends (90/379, 23.7%). As multiple responses were permitted for this item, percentages do not sum to 100%. Participants who identified healthcare providers as their primary source of information demonstrated significantly higher knowledge scores compared with those relying on non-professional sources (p < 0.05). The distribution of reported information sources is shown in Figure 2.

Statistical associations

A statistically significant but modest positive correlation was observed between educational level and folic acid knowledge scores (Spearman's r = 0.219; p = 0.001), indicating that higher educational attainment was associated with better knowledge. No statistically significant associations were found between folic acid knowledge and previous pregnancy history (p = 0.124), age (p = 0.083), or marital status (p = 0.156).

**Figure 3:**
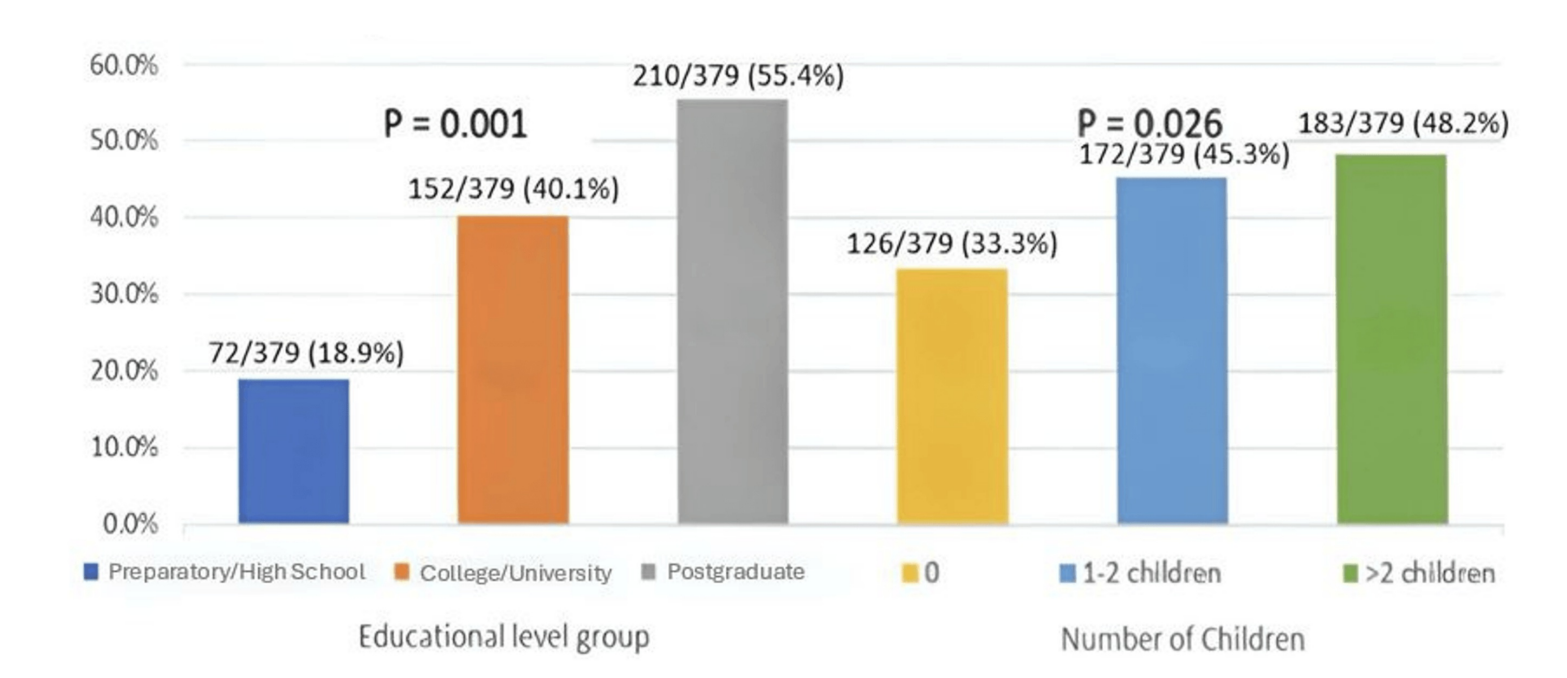
Proportion of women achieving high knowledge scores according to educational level and number of children (n = 379).

The proportion of participants achieving high knowledge scores according to educational level and number of children is presented in Figure 3. These findings suggest that educational exposure plays a more influential role in shaping folic acid knowledge than reproductive experience alone.

## Discussion

This study provides important insights into the awareness and understanding of folic acid among women of childbearing age in the UAE, a country characterized by a diverse population and well-established maternal and primary healthcare services. While overall awareness was high (87.9%), notable gaps appeared in participants' understanding of the specific preventive role of folic acid and the importance of starting supplementation before conception. These findings reflect a pattern previously reported across the region [5,7,9-12].

Comparison with previous studies

The results align with earlier UAE-based work, such as the Knowledge, Attitudes, and Practices (KAP) study by Al-Hossani et al. [5], which demonstrated that although many pregnant women were familiar with folic acid, fewer understood its role in preventing NTDs. Studies in Saudi Arabia have reported a disconnect between general awareness and correct knowledge or adherence [7,9-12]. Recent research from Saudi Arabia has further shown that even where awareness exceeds 90%, only a minority of women initiate folic acid supplementation at the recommended time [9,10]. Additional regional investigations have demonstrated variability across subgroups, particularly by educational level, age, and pregnancy status, with women of lower educational attainment consistently showing lower knowledge levels [13-15].

Determinants of knowledge

Education emerged as a significant predictor of folic acid knowledge in this study, consistent with earlier findings in Middle Eastern populations [6]. Higher educational attainment is often associated with improved health literacy and a greater likelihood of engaging with preventive health information. Interestingly, reproductive history did not correlate with higher knowledge levels, a finding also observed in studies from Saudi Arabia [12] and Jordan [16,17]. These findings suggest that educational exposure plays a more influential role in shaping folic acid knowledge than reproductive experience alone. Prior pregnancy experience, therefore, appears insufficient to ensure correct understanding, particularly when counselling is inconsistent or initiated only after conception. This highlights the importance of structured health education and proactive communication strategies rather than reliance on experiential learning.

Role of healthcare providers and media

Healthcare professionals were the most frequently cited source of information, which is consistent with previous regional studies [8]. While this is encouraging, reliance solely on clinical encounters may be inadequate, as many women seek medical advice only after confirming pregnancy. Observational evidence from a cross-sectional study conducted in Saudi Arabia suggests that women exposed to both clinical counselling and broader public health messaging demonstrated higher knowledge and better supplementation practices [9]. Broader engagement through mass media, including television, radio, social media platforms, and educational institutions, has also been shown to enhance awareness and reach women who might not otherwise receive structured guidance [11].

Knowledge gaps and behavioral implications

Despite the high overall awareness observed in this study, only 38.7% of participants recognized the importance of taking folic acid before conception. This gap is clinically significant because neural tube closure occurs within the first four weeks of pregnancy [1]. Similar misconceptions regarding timing and benefits of folic acid supplementation have been widely reported in Middle Eastern and international studies [18,19]. Public health initiatives promoting periconceptional folic acid supplementation, particularly when combined with food fortification and structured preconception counselling, are widely recognized as effective strategies for reducing NTDs. Ensuring that women in the UAE receive timely and accurate information, particularly prior to pregnancy, may be important for improving maternal and child health outcomes.

Limitations and future directions

This study has several limitations. Although the questionnaire was adapted from previously published instruments and reviewed for content validity, formal psychometric validation was not performed. Future studies should consider conducting comprehensive validation procedures, including reliability testing, to strengthen measurement accuracy. The use of an online survey may have introduced sampling bias, particularly among women with limited digital access or lower literacy levels. Additionally, restricting participation to women who could read Arabic or English may have limited the inclusion of certain expatriate subgroups within the UAE's multilingual population, potentially affecting representativeness. As with most self-reported data, participants may have overestimated their knowledge or adherence to folic acid recommendations. Future research should employ probability-based or clinic-based recruitment strategies to enhance generalizability, include more diverse and underrepresented groups, and explore qualitative approaches to better understand cultural and behavioral determinants of supplementation practices. Investigating whether increased knowledge translates into sustained behavioral change and identifying barriers to timely supplementation initiation would also be valuable.

Implications for public health

The findings suggest a potential need for comprehensive, culturally tailored public health initiatives to improve folic acid awareness and correct supplementation practices in the UAE. A multipronged approach integrating clinical counselling with community outreach, school-based education, university initiatives, and targeted media campaigns may be particularly effective. Incorporating folic acid education into premarital counselling, family planning services, and routine primary care could further enhance timely supplementation. Given the strong global evidence supporting folic acid fortification and structured preconception counselling, broader implementation of these strategies in the UAE may contribute to reducing the burden of preventable NTDs and improving maternal and child health outcomes.

## Conclusions

This study demonstrates that although general awareness of folic acid among women of childbearing age in the UAE is high, detailed knowledge regarding its role in preventing NTDs and the appropriate timing of supplementation remains insufficient. Fewer than half of the participants correctly identified that folic acid should be initiated before conception. Higher educational attainment was significantly associated with better knowledge, whereas reproductive history alone was not. Healthcare providers were the primary source of information, underscoring their critical role in reinforcing preconception counselling. Targeted educational strategies emphasizing early supplementation may help improve knowledge and reduce the risk of NTDs in the UAE.

## Appendices

**Table 2:** Questionnaire used to assess awareness and knowledge of folic acid among women of childbearing age in the UAE (English version) AED: United Arab Emirates Dirham; UAE: United Arab Emirates

Section	Question	Response options
Sociodemographic characteristics	What is your age?	18-24 years
		25-29 years
		30-34 years
		35-39 years
		40-44 years
		45-49 years
	What is your nationality?	UAE national
		Non-UAE national
	What is your current level of education?	Primary school or lower
		Preparatory/high school
		College/university
		Postgraduate
	What is your employment status?	Employed
		Unemployed
		Retired
	What is your family income per month?	<5,000 AED
		5,000-19,999 AED
		20,000-30,000 AED
		>30,000 AED
	Do you have access to health insurance?	Yes
		No
	What is your marital status?	Single
		Married
		Divorced
		Widowed
Reproductive history	Are you currently pregnant or trying to conceive?	Currently pregnant
		Planning to conceive
		Not planning to conceive
		Does not apply
	If you were pregnant before, when was your last pregnancy?	<5 years ago
		5-10 years ago
		10-15 years ago
		>15 years ago
	If you have children, how many do you have?	One
		Two
		Three
		More than three
Awareness of folic acid	Have you heard about folate or folic acid before?	Yes
		No
	If yes, where did you hear about folic acid? (Multiple responses allowed)	Media
		School/university
		Doctors
		Other health professionals
		Relatives
		Books/magazines
		Other
Information provided to participants	What is folate and folic acid?	"Folate is a vitamin found naturally in food, and folic acid is a synthetic form of folate."
	To what extent do doctors emphasize the importance of folic acid?	Likert scale (0-5)
	How much do you know about folic acid?	Likert scale (0-5)
Knowledge and attitudes	Who should take folic acid supplements? (Multiple responses allowed)	Women planning pregnancy
		Pregnant women
		Postpartum women
		Children
		Elderly
		I do not know
	How important is folic acid intake for pregnant women or those planning pregnancy?	Likert scale (0-5)
	When should women take folic acid supplements? (Multiple responses allowed)	Always
		Before pregnancy
		During pregnancy
		After pregnancy
		I do not know
	How knowledgeable are you about the consequences of folic acid deficiency during pregnancy?	Very knowledgeable
		Somewhat knowledgeable
		Not knowledgeable
		I do not know
	What is the main benefit of folic acid during pregnancy?	Prevents neural tube defects/congenital abnormalities
		Helps fetal growth
		Helps heart development
		I do not know
		Other
Practices and beliefs	Do you believe more education about folic acid is needed?	Yes
		No
	Are you against taking pregnancy supplements?	Yes
		No
		Other
	If yes, please specify the reason	Open-ended
	Preferred source of vitamins/minerals during pregnancy	Organic sources
		Pharmacological sources
		Homeopathy
		Other
	Which other vitamins/minerals are important during pregnancy? (Multiple responses allowed)	Iron
		Calcium
		Vitamin D
		Vitamin C
		Vitamin K
		Vitamin A
		Omega-3 fatty acids
		I do not know
	Are you afraid of multivitamin overdose?	Yes
		No
	Which foods do you consume that contain folate/folic acid? (Multiple responses allowed)	Green vegetables
		Other vegetables
		Grains/legumes
		Citrus fruits
		Other fruits
		Chickpeas
		Dried beans
		Peas
		Nuts
		Milk
		I do not know
